# Molecular Functions of Thyroid Hormones and Their Clinical Significance in Liver-Related Diseases

**DOI:** 10.1155/2013/601361

**Published:** 2013-06-26

**Authors:** Hsiang Cheng Chi, Cheng-Yi Chen, Ming-Ming Tsai, Chung-Ying Tsai, Kwang-Huei Lin

**Affiliations:** ^1^Department of Biochemistry, School of Medicine, Chang-Gung University, Taoyuan 333, Taiwan; ^2^Department of Nursing, Chang-Gung University of Science and Technology, Taoyuan 333, Taiwan

## Abstract

Thyroid hormones (THs) are potent mediators of several physiological processes, including embryonic development, cellular differentiation, metabolism, and cell growth. Triiodothyronine (T_3_) is the most biologically active TH form. Thyroid hormone receptors (TRs) belong to the nuclear receptor superfamily and mediate the biological functions of T_3_ via transcriptional regulation. TRs generally form heterodimers with the retinoid X receptor (RXR) and regulate target genes upon T_3_ stimulation. Research over the past few decades has revealed that disruption of cellular TH signaling triggers chronic liver diseases, including alcoholic or nonalcoholic fatty liver disease and hepatocellular carcinoma (HCC). Animal model experiments and epidemiologic studies to date imply close associations between high TH levels and prevention of liver disease. Moreover, several investigations spanning four decades have reported the therapeutic potential of T_3_ analogs in lowering lipids, preventing chronic liver disease, and as anticancer agents. Thus, elucidating downstream genes/signaling pathways and molecular mechanisms of TH actions is critical for the treatment of significant public health issues. Here, we have reviewed recent studies focusing on the roles of THs and TRs in several disorders, in particular, liver diseases. We also discuss the potential therapeutic applications of THs and underlying molecular mechanisms.

## 1. Introduction

Thyroid hormones (THs), particularly triiodothyronine (T_3_), are potent regulators of multiple physiological activities, including cellular metabolic rate, heart and digestive functions, muscle function, brain development, and bone maintenance [1, 2]. In addition to their crucial roles in maintaining cellular homeostasis, THs can cause multiple disorders, including cardiovascular disease [3, 4], diabetes mellitus [5, 6], and chronic liver disease [7–9], when their levels in the body are out of balance. The liver is a typical target organ of THs. Equal amounts of thyroid hormone receptor *α*1 and *β*1 (TR*α*1 and TR*β*1) proteins are expressed in human hepatocytes [10]. Earlier studies reported that treatment with T_3_ analogs prevents hepatic steatosis and hepatitis [11–16]. Additionally, THs have potential therapeutic applications in hepatitis B and C [17, 18]. Interestingly, T_3_ and TRs also play important roles in the pathogenesis of hepatocellular carcinoma (HCC). For instance, v-erbA, a mutant form of TR lacking ligand-binding ability, triggers HCC development in transgenic mice [19, 20]. Moreover, our group and other researchers have shown that cloned TR*α* and TR*β* genes are truncated or mutated at high frequencies in human HCCs [21–23]. Several groups, including ours, have focused on the roles of T_3_ and TRs in liver disease, especially liver cancer. DNA microarrays have been widely employed to identify genes regulated by T_3_ in hepatoma cell lines and provide an effective means for elucidating the roles of T_3_/TRs in human HCC. The results obtained to date collectively indicate that T_3_ and TR influence hepatoma cell growth, metabolism, apoptosis, and metastasis [23–28], suggesting powerful therapeutic potential in clinical applications [29].

In the current report, we review studies by our research group and other investigators on the roles of THs and TRs in liver diseases, particularly HCC. Elucidating the molecular basis for the effects of T_3_/TR on hepatocytes and hepatoma may facilitate the design of improved strategies for preventing or curing liver-related diseases ranging from steatosis to HCC.

## 2. Actions of Thyroid Hormones and Thyroid Hormone Receptors

The physiological actions of THs affect almost every organ system. Clinically, these effects are observed as changes in metabolic rate, altered lipid metabolism, and characteristic effects on cardiovascular development [30–32]. Under physiological conditions, T_4_ is the main hormone secreted into the bloodstream by the thyroid gland. Conversion of T_4_ to the more active form, T_3_, is regulated in extrathyroidal tissue through the selenoprotein enzyme system. Expression levels and activities of type I and type II deiodinases (D1 and D2) vary among tissues, leading to tissue-specific differences in the levels of circulating T_3_ and active hormone available for binding to nuclear receptors. Conversely, type III deiodinase (D3), which converts T_4_ and T_3_ to the comparatively inactive forms, reverse triiodothyronine (rT_3_) and 3,3′-diiodothyronine (T_2_), respectively, is responsible for suppression of hormone activity [33]. Both T_3_ and T_4_ act via TRs. However, the TR binding affinity of T_4_ is considerably lower than that for T_3_. According to the genomic action of thyroid hormone, T_3_ binding with TRs located on thyroid hormone response elements (TREs) of promoter regions induces target gene expression at the transcriptional level [20, 34, 35].

### 2.1. Thyroid Hormone Receptors

TRs belong to the nuclear receptor superfamily and act as T_3_-inducible transcription factors. TRs are encoded by two genes, THRA and THRB, located at separate loci [36]. The THRA gene, located on chromosome 17, encodes one functional T_3_-binding TR*α*1 and two dominant-negative splice variants, TR*α*2 and TR*α*3 [37], that lack T_3_ binding ability [38]. TRΔ*α*1 and TRΔ*α*2 are truncated variants transcribed from an internal promoter located within intron 7. These truncated forms lack DNA-binding domains, but retain T_3_-binding competence [39]. TR*α*1 and TR*α*2 are highly expressed in the brain, with lower abundance in the kidneys, skeletal muscle, lungs, heart, and testes [40].

THRB genes are located on chromosome 3 and encode three functional T_3_-binding TR*β* isoforms (*β*1, *β*2, and *β*3) [37]. Interestingly, the truncated TR*β* variant, TRΔ*β*3, lacks the DNA-binding domain but retains T_3_ binding activity and acts as a dominant-negative antagonist [37]. TR*β*1 is widely expressed in all tissues, but is particularly prominent in brain, thyroid, liver, and kidney, whereas TR*β*2 is predominantly expressed in a tissue-specific manner in the anterior pituitary, hypothalamus, retina, developing brain, and inner ear [36, 37, 41, 42]. A novel human carboxy-terminal spliced variant of TR*β*1 that lacks T_3_-binding ability and acts as a dominant-negative isoform, designated TR*β*4, has recently been cloned. TR*β*4 is widely expressed in all human tissues, but is highly abundant in testis and skeletal muscle [43]. Interestingly, whereas human TR*α*1, TR*α*2, and TR*β*1 are abundant in different tissues, none is highly expressed in the liver, the major TH target organ [38].

### 2.2. Transcriptional Activities of T_3_/TRs

**Figure 1:**
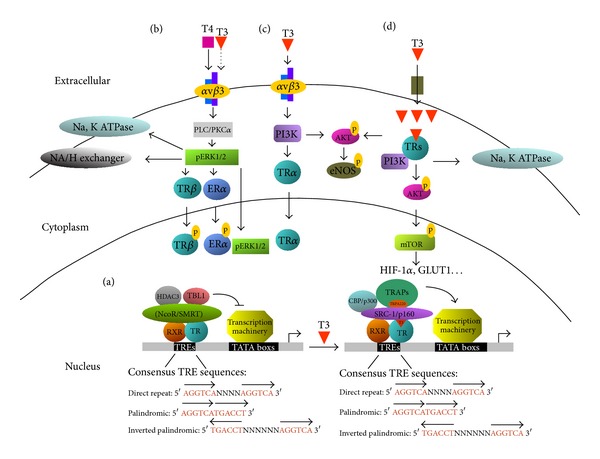
Molecular actions of THs and TRs. The actions of THs are mediated via genomic and nongenomic effects.

The protein structure of TRs, similar to that of other nuclear receptors, contains modular functional domains, including an N-terminal A/B domain, a DNA-binding domain that recognizes TREs within the target gene promoter, a hinge region (D), and a C-terminal ligand-binding domain (E) responsible for dimerization with other nuclear receptors or interactions with transcriptional coregulators [36, 44–47]. The TR exerts its transcriptional activity as a homodimer or as a heterodimer with other nuclear receptors, including retinoid X receptor (RXR) and other retinoic acid receptor subtypes, and vitamin D receptors (VDR). RXR is the general partner of several nuclear receptors in the regulation of target genes [48]. TRs generally heterodimerize with RXRs and localize to TREs within the promoter regions of target genes (Figure 1(a)). The TR/RXR heterodimer exhibits the highest T_3_ binding affinity and remains stable during ligand binding. The known requirement of RXR for maximal transcriptional activation by TRs highlights the crucial role of these interactions [49, 50]. 

TREs within promoter regions of T_3_ target genes generally contain a core consensus “half-site” with the sequence (A/G)GGT(C/A/G)A. A typical TRE contains two half-site sequences in palindromic, direct-repeat, or inverted-repeat arrangements that are recognized by TRs (Figure 1(a)) [45].

In the absence of T_3_, TRs usually act as transcriptional repressors. Upon T_3_ binding, TRs undergo a conformational change that causes dissociation of their corepressors, such as nuclear receptor corepressor (NCoR) or itshomolog silencing mediator of retinoic and thyroid receptor (SMRT), allowing induction of target gene expression (Figure 1(a)). NCoR and SMRT corepressor-mediated TR functions have been extensively characterized [36]. Specifically, NCoR and SMRT display histone deacetylase activity and serve as a platform for repressor complex-mediated chromatin remodeling. In addition, both corepressors are associated with histone deacetylase 3 (HDAC3) and other proteins, such as transducin-like protein (TBL1), forming large repressor complexes [51] that suppress transcription via activation of HDAC3 or TBL1.

The conformational change in TRs induced by T_3_ binding leads to the recruitment of transcriptional coactivators of target gene transcription. The steroid hormone receptor coactivator (SRC) and p160 family members that interact with ligand-bound TR to facilitate activation of T_3_ target genes are well characterized [52]. SRC-1 utilizes its histone acetyltransferase activity to loosen chromatin structures, thereby promoting nuclear receptor transactivation. In addition to the SRC/p300 and p160 families, the TR-associated protein (TRAP) family stimulates the transcriptional activities of TRs [53]. Members of this family form a large complex with TRs that does not exhibit intrinsic histone acetyltransferase activity, but enhances TR transactivation ability. Combinatorial association of TR with different coregulators may contribute to the differential responses necessary for the appropriate expression of target genes. Moreover, the multiple functions of T_3_/TR are triggered via posttranslational modification of either TRs or their coregulators in response to external stimuli [45, 54].

### 2.3. Nongenomic Effects of Thyroid Hormones

Activities of THs triggered in the plasma membrane or cytoplasm that transmit T_3_ signals through nuclear TRs are termed ‘‘nongenomic effects”. Recently, the plasma membrane protein, integrin *α*v*β*3, was characterized as membrane-bound TR and shown to mediate T_3_ signals through a binding domain for iodothyronine [55]. This receptor contains the Arg-Gly-Asp (RGD) recognition region responsible for interactions with extracellular matrix (ECM) ligands [55]. Upon binding to T_3_ and T_4_, the mitogen-activated protein kinase/extracellular signal-regulated kinase (MAPK/ERK 1/2) pathway is activated, regulating multiple cellular physiological effects [56] (Figure 1(b)). THs interact with integrin receptor, *α*v*β*3, to stimulate activation and trafficking of activated ERK 1/2 through phospholipase C (PLC) and protein kinase C*α* (PKC*α*). The binding affinity of T_4_ to this site was higher than that of T_3_, and T_4_-activated ERK 1/2 modulates intracellular protein trafficking of estrogen receptor a (ER*α*) and TR*β*1 from the cytoplasm to nucleus. Additionally, activated ERK 1/2 induces the sodium proton exchanger (Na+/H+) and increases activity of the sodium pump (Na, K-ATPase). Stimulation of the phosphatidylinositol 3-kinase (PI3K)/Akt/protein kinase B (PKB) pathway is another effector-mediated nongenomic action of TH. T_3_-induced PI3K activity may be initiated by binding to membrane-bound integrin *α*v*β*3 or via a mechanism that is cytoplasmic in origin (Figures 1(c) and 1(d)) [57]. In Figure 1(c), the bioactive form of TH, T_3_, but not T_4_, interacts with a T_3_-specific binding domain of integrin *α*v*β*3 to activate the PI3K signal pathway via stimulation of Src kinase. T_3_-mediated PI3K activation also leads to trafficking of TR*α*1 from the cytoplasm to the nucleus and increased target gene expression, such as HIF-1*α*. In Figure 1(d), in the cytoplasm, T_3_ rapidly induces the PI3K pathway and initiates downstream gene transcription. T_3_-liganded TR*β*1 in the cytoplasm interacts with the PI3K regulatory subunit, p85*α*, and induces Akt phosphorylation. Activated Akt subsequently phosphorylates and activates nuclear mTOR and downstream genes. Additionally, TR*α*1 interacts with the p85*α* subunit of PI3K in a T_3_-dependent manner, leading to activation of Akt and endothelial nitric oxide synthase (eNOS). T_3_/T_4_ binding to the recognition site of integrin *α*v*β*3 activates PI3K, stimulating shuttling of TR*β*1 from the cytoplasm to the nucleus and increasing expression of target genes such as hypoxia inducible factor-1*α* (HIF-1*α*) [58]. TH additionally induces phosphorylation and nuclear translocation of other potent factors responsible for several cellular functions (Figure 1(c)) [59]. For instance, TH-activated ERK1/2 has been shown to promote the expression or activity of estrogen receptor-*α* (ER*α*), signal transducer and activator of transcription-3 and -1*α* (STAT3 and STAT1*α*), and several TR-associated proteins [60–65]. T_3_ activates PI3K-Akt/PKB, leading to activation of the nuclear mammalian target of rapamycin (mTOR)-p70S6K cascade and sequential induction of several HIF-1*α* target genes, including glucose transporter 1 (GLUT1), platelet-type phosphofructokinase (PFKP) and monocarboxylate transporter 4 (MCT 4) (Figure 1(d)) [66–68], which play critical roles in cellular metabolism. In endothelial cells, TH-mediated Akt phosphorylation induces endothelial nitric oxide synthase (eNOS) activity and thereby regulate vascular function [69, 70]. Additionally, TR*β*1 has been shown to modulate the activity of Na, K-ATPase via stimulation ofPI3K or ERK1/2. For instance, PI3K signaling causes slowed deactivation of KCNH2 (potassium voltage-gated channel, subfamily H, member 2) in the plasma membrane of pituicytes [71–73].

## 3. Roles of Thyroid Hormones and Thyroid Receptors in Liver Diseases

### 3.1. Alcoholic Fatty Liver Disease

Alcohol abuse and alcohol-induced liver diseases (ALD) pose a major public health problem worldwide. ALD is possibly the main cause of death among individuals with severe alcohol abuse and accounts for about 3.8% of global mortality [74]. Alcohol consumption-related liver diseases are divided into three categories: fatty liver, alcoholic hepatitis, and cirrhosis [75, 76]. The liver plays a major role in alcohol metabolism and represents the primary site of ethanol-related damage. The liver is the major target organ of THs, and cellular levels of these hormones are closely associated with alcohol-related disease [77]. Moreover, a significant reduction in thyroid gland volume has been reported in patients with alcohol-dependent liver diseases. For these patients, low serum T_3_ and free T_3_ index values at admission accompanied by a normal serum T_4_, free T_4_ index, and thyroid stimulating hormone (TSH) levels appear indicative of severe liver dysfunction and increased mortality risk [78–81]. Further studies have confirmed a reduction of circulating free T_3_ (fT_3_) in subjects with alcoholic hepatitis and cirrhosis. The absence of abnormalities in subjects with dyslipidemia, despite ethanol intake similar to that in the other groups, and correlations between fT_3_ and liver function tests suggest that changes in fT_3_ reflect the severity of underlying liver disease [82]. In addition to alcohol-related diseases, acute alcohol withdrawal or long-term abstinence periods after alcohol dependence has been reported to reduce peripheral TH levels [83]. Furthermore, a decrease in free TH levels may be a result of heavy alcohol consumption or a marker of alcoholism [77].

### 3.2. Nonalcoholic Fatty Liver Disease

Since 1845 when alcohol-related liver disease was first identified, fatty liver disease (FLD) was mainly attributed to the excessive consumption of alcohol. In 1981, nonalcoholic fatty liver disease (NAFLD), representing a spectrum of hepatic diseases ranging from simple steatosis to steatohepatitis, advanced fibrosis and cirrhosis, was first described. Nonalcoholic steatohepatitis (NASH) describes a liver disease histologically similar to alcoholic hepatitis that occurs without abuse of alcohol, referring to a stage within the spectrum of NAFLD [84, 85].

**Figure 2:**
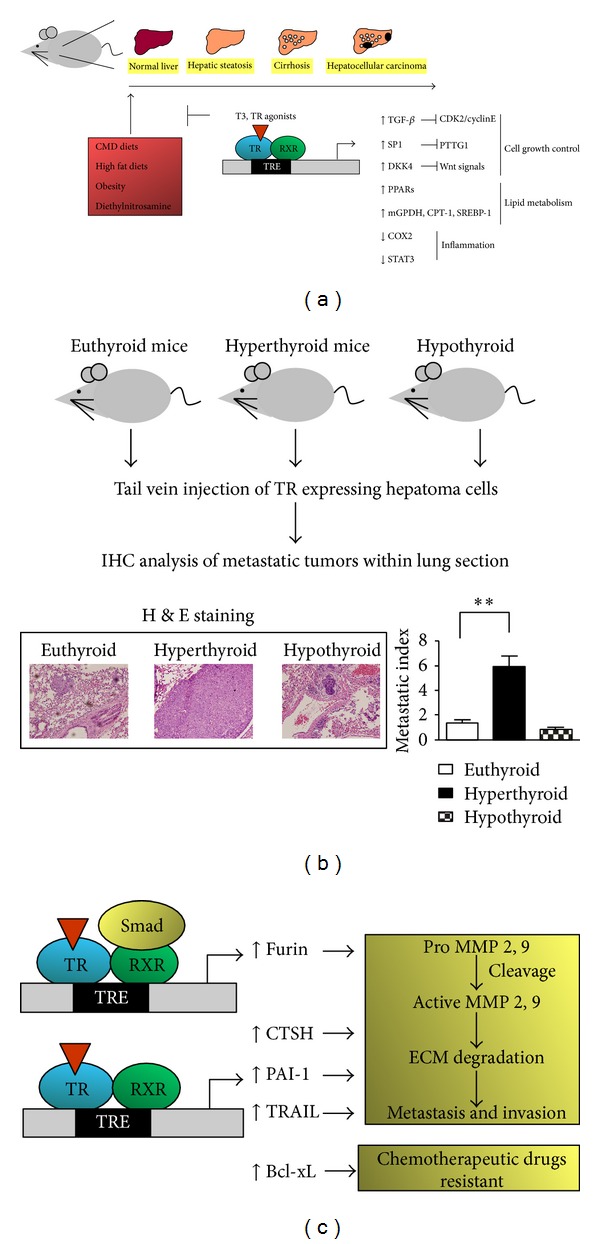
Molecular functions of T_3_/TR in liver diseases. T_3_/TR suppresses several liver diseases ranging from simple steatosis to HCC, but also promotes tumor metastasis and chemotherapeutic resistance.

Despite the high prevalence of NAFLD and its potential for serious sequelae, the underlying etiologic factors that determine disease progression remain poorly understood; therefore, effective therapeutic strategies need to be further explored. Recent metabolic studies in humans and animals have demonstrated a close association with insulin resistance and several features of the metabolic syndrome. Moreover, over 90% of patients with NAFLD present at least one feature of metabolic syndrome, and diabetes or insulin resistance may accelerate the entire pathological spectrum [85]. NAFLD may be triggered by secondary effects of medications (e.g., corticosteroids, tamoxifen), nutritional causes (e.g., rapid weight loss) or metabolic disorders (e.g., lipodystrophy or dysbetalipoproteinemia) [86, 87]. Remarkably, TH, a potent regulator of cellular metabolism, has potential therapeutic application in NAFLD prevention (Figure 2(a)). Perra et al. [12] developed experimental models to investigate the biochemical changes in NAFLD and NASH. High-fat choline-methionine-deficient (CMD) diets were employed to induce dyslipidemia, hepatocyte injury, fibrosis, cirrhosis, oxidative DNA damage, and HCC in rodents [88–90]. Following 10 weeks of a CMD diet in rats, administration of T_3_ or an analog for only 1 week led to a dramatic reduction in triglyceride (TG) accumulation in the liver. Moreover, pathways associated with inflammation-related genes, such as STAT3 and cyclooxygenase-2 (COX-2), were suppressed (Figure 2(a)), reducing the severity of liver injury, as determined based on serum transaminase levels. Notably, the therapeutic effects of T_3_ were sharedby GC-1, a selective agonist of TR*β* [91]. Animal experiments revealed that GC-1 causes an even greater reduction in TG levels than that produced by equimolar doses of T_3_. Moreover, these effects were elicited at doses that induced no significant side effects on heart rate, muscle loss, or increase in the overall catabolic state [91–93]. These findings support the potential therapeutic application of TH in steatosis prevention (Figure 2(a)).

MB07811 is an orally active HepDirect prodrug of MB07344, a liver-targeted TR*β* agonist that has been tested in normal rodents and rodent models of NAFLD [13]. MB07811 markedly reduces hepatic steatosis as well as plasma free fatty acid (FFA), TGs and aspartate transaminase (ALT) levels; it may also contribute to increased metabolic rate in liver, specifically, an increase in mitochondrial and fatty acid *β*-oxidation cycles. Expression levels and activities of genes responsible for energy production or lipid metabolism, including hepatic mitochondrial glycerol-3-phosphate dehydrogenase (mGPDH), carnitine palmitoyl transferase 1 (CPT-1), and sterol regulatory element binding protein-1c (SREBP-1c), are increased following MB07811 treatment [13, 94] (Figure 2(a)).

In addition to analogs of THs, such as GC-1 and MB07811, target genes of TR that provide therapeutic benefits to hyperlipidemic patients with concomitant NAFLD have also been investigated. In these experiments, signals downstream of THs in the livers of rats fed a high-fat diet were analyzed.

TH was shown to regulate the peroxisome proliferator-activated receptors, PPAR*α*, PPAR*γ*, and PPAR*δ*, which target genes for enzymes such as acyl-CoA oxidase, stearoyl-CoA, and adipose triglyceride lipase that are important in lipid metabolism and lipid droplet turnover (Figure 2(a)). Moreover, apolipoprotein B (apo B), the major protein component of very low-density lipoproteins (VLDLs), was found to be regulated by THs. These findings suggest that, in addition to stimulating lipid oxidation pathways, TH suppresses the pathways leading to lipid storage in lipid droplets and promotes lipid mobilization from lipid droplets and secretion as VLDLs [95].

In brown adipose tissue, TH activation via type II deiodinase is necessary for adaptive thermogenesis. However, the D2 knockout (D2KO) mice exhibit both the phenotypes of impaired thermogenic responses to cold and liver steatosis. Liver steatosis of D2KO mice was induced via increased unsaturated fatty acid uptake, impaired *β*-oxidation, and decreased VLDL secretion [96].

Fibroblast growth factor 21 (FGF21), a member of the endocrine fibroblast growth factor (FGF) subfamily, has the ability to improve insulin sensitivity and glucose clearance, reduce plasma TG concentrations, and promote resistance to weight gain in mice fed a high-fat diet [97]. Both *in vitro* and *in vivo* experiments have demonstrated that the effects of T_3_- and TR*β*-specific agonists are due, at least in part, to induction of hepatic FGF21 and possible suppression of FGF21 in white adipose tissue, providing a mechanistic basis for TH activity in NAFLD therapy [98].

In addition to animal experiments, cross-sectional studies have been conducted. Subclinical hypothyroidism, even in the range of upper normal TSH levels, was found to exhibit a dose-dependent relationship to NAFLD [99]. Moreover, hypothyroidism was found to be closely associated with NAFLD independently of known metabolic risk factors [99]. Similar results have consistently been obtained by other groups. Serum T_3_ level appeared to be significantly correlated with serum bilirubin, albumin, and prothrombin time in patients with liver cirrhosis and chronic hepatitis [8], whereas serum fT_4_ concentrations were inversely associated with hepatic steatosis [100]. Serum TSH levels were significantly higher in the nonalcoholic fatty liver than in the normal group. Thyroid function tests additionally confirmed the existence of several thyroid functional abnormalities in patients with chronic liver disease. However, in euthyroid patients, TSH level was not an independent risk factor, and changes in TSH levels did not influence the prevalence of NAFLD [8, 9, 101].

Several coregulators act in cooperation with TR to control hepatic TG levels and maintain hepatic insulin sensitivity. For instance, disruption of SMRT and nuclear receptor interactions leads to insulin resistance and fatty liver [102]. A recent study showed that ligand-dependent corepressor (LCOR) decreases hepatic TG levels in ob/ob and diet-induced obese mice cooperatively with TR [103]. NCoR-HDAC3 interactions regulate T_3_-mediated autophagy and lipid metabolism *in vivo* [104]. Thus, coregulators, such as LCOR, may present potential targets for the effective treatment of hepatic steatosis.

Conversely, TH/TR exerts preventive effects on steatohepatitis. In mice with high-fat diet-induced NAFLD, TR*α*-null animals are leaner and exhibit greater whole-body insulin sensitivity than their wild-type counterparts. Thus, the loss of TR*α* protects against high-fat diet-induced hepatic steatosis as well as hepatic and peripheral insulin resistance. Accordingly, inhibition of TR*α* represents a novel pharmacologic strategy for the treatment of NAFLD, obesity, and type II diabetes [105–107].

### 3.3. Liver Cancer

Liver cancer is one of the most prevalent causes of tumor-related mortality and the fifth-most common cancer type worldwide [108]. HCC represents the most common histological primary liver malignancy subtype and is the leading cause of cancer-related death in Asia and Africa [109]. Epidemiological studies indicate that liver cancer is linked to high mortality rates from liver cirrhosis, and the majority (80–90%) of HCCs develop from cirrhotic livers [110]. Moreover, the pathogenesis of HCC is characterized by multi-step carcinogenic process arising in the liver. Globally, chronic infection with hepatitis B (HBV) or hepatitis C (HCV) virus is a major risk factor for HCC. HBV infection accounts for approximately 50% of HCC cases in the majority of Asian countries [111]. Although HBV infection can cause HCC in the absence of cirrhosis, the majority (70–80%) of patients with HBV-related HCC display cirrhosis. Patients at risk of infection with HBV include older males infected over a long period of time. Other risk factors include significant consumption of alcohol, tobacco smoking, exposure to aflatoxin, and coinfection with HCV [111, 112]. HCV is a particularly significant risk factor for HCC in Japan, but it is also important elsewhere. Records show that 80% to 90% of patients with HCC are infected with HCV in Japan, 44% to 66% in Italy, and 30% to 50% in the United States. The risk factors for HCV infection are advanced age and male gender, coinfection with HBV, and possibly, metabolic disorders such as obesity or diabetes [111, 113, 114].

A high proportion of HCC patients suffer from severe metabolic disorders, chronic liver diseases, or HBV and HCV infections. Moreover, THs and TRs appear to have preventive activities against chronic liver cirrhosis. A previous study revealed that the serum levels of THs are decreased in HBV- and HCV-infected patients [17]. HCC is accompanied by a significant increase in serum rT_3_ (inactive form of T_3_) levels in patients with low-grade HCV-related liver cirrhosis [115]. Thus, it is important to determine the functions of TH signaling in HCC.

In 1986, v-erbA, a mutant form of TR devoid of ligand-binding ability borne by the avian erythroblastosis virus, which causes erythroleukemia, was identified and subsequently shown to trigger HCC in transgenic mice [19, 20]. A correlation between TR and carcinogenesis was further reported.

Accumulating evidence from recent studies supports an association between aberrant TR regulation (or TRmutation) and human neoplasia [116]. Moreover, several experiments by our group and other researchers have shown that mutated or truncated forms of TR*α* and TR*β* are expressed at high frequencies in human HCCs [21–23, 117, 118]. These mutant forms of TRs display loss of transcriptional activity, defects in the release and binding of ligand-driven corepressor, and may further act as dominant-negative forms of TRs.

In rat models of HCC created by a combination of diethylnitrosamine (DEN) and partial hepatectomy, 2-acetylaminofluorene (2-AAF) or CMD diet, T_3_/TR was shown to suppress the carcinogenic process through induction of a preneoplastic hepatocyte differentiation program (Figure 2(a)). Moreover, T_3_ acts as a mitogenic factor to induce hepatocyte proliferation without causing cell death; the resulting acceleration of proliferation rate is associated with anticancer effects, including nodule regression, in the carcinogen-induced HCC model [119–122]. A recent epidemiological study suggested that long-term hypothyroidism is associated with the incidence of HCC, independent of other HCC risk factors [123]. Hypothyroidism is characterized by insufficient production of thyroid hormone and inappropriate TR action.

The above studies collectively indicate that disruption of TH signaling causes neoplasia, particularly HCC. Therefore, an analysis of T_3_/TR target genes in HCC is necessary to elucidate the actions of T_3_/TR that influence either the incidence of liver cancer or HCC progression. Previously, we employed cDNA microarrays to analyze target genes following T_3_ treatment in wild-type TR-expressing hepatoma cells. T_3_ appears to influence hepatoma cell proliferation, metabolism, apoptosis, and metastasis via regulation of downstream genes [24, 25, 124, 125]. These T_3_ target genes also mediate several potent signaling pathways in HCC progression.

Among the genes targeted by THs is transforming growth factor-beta (TGF-*β*), a potent regulator of cell growth and proliferation. TGF-*β* has been shown to block the growth of numerous cell types, including hepatoma cells [126].

Previous experiments by our group showed that TGF-*β* is upregulated by T_3_ at the mRNA level, leading to inhibition of cell proliferation [125]. Additionally, T_3_ represses the expression of several cell cycle regulators, including cyclin-dependent kinase 2 (CDK2), cyclin E, and phosphorylated pRb (ppRb). T_3_ exerts an anti-growth effect in HCC cells via TGF-*β* stimulation (Figure 2(a)). However, the role of the TGF-*β* signaling in HCC is controversial at present. Paradoxically, TGF-*β* has been shown to both inhibit cell growth and promote tumor cell metastasis by inducing epithelial-mesenchymal transition (EMT) [127]. Furthermore, the molecular classification of HCC is based on the clinical significance of the genes embedded in the TGF-*β* expression signature [128]. The precise functions of TGF-*β* signaling in HCC progression may reflect the different microenvironments produced via tumor-stromal interactions.

In another study, we showed that the TH promotes hepatoma cell invasion in cooperation with TGF-*β* via regulation of several target genes (e.g., furin). Furin, a member of the protease family of proprotein convertase subtilisin/kexins (PCSKs), activates precursor proteins by specifically cleaving a motif of basic paired amino acids, RXK/RR, at the carboxyl terminus [129], converting inactive precursor growth factors into their mature forms, which promote tumor growth. In general, PCSKs are ubiquitously expressed and mediate numerous pathophysiological processes [129, 130], such as the conversion of inactive precursor proteins into biologically active products that are vital for several biological functions. Among PCSKs are members of the matrix metalloproteinase (MMP) family, which are critical mediators of tumor cell metastasis [130]. Our experiments demonstrated that T_3_ cooperates with TGF-*β* and its components, Smad3/4, to upregulate furin expression. Additionally, T_3_-induced furin activation was mediated by the ERK pathway. Induction of furin expression involved crosstalk among T_3_, TGF-*β*, and ERK pathways. Furin activation led to an aggressive phenotype in hepatoma cells, characterized by enhanced invasion and metastasis (Figures 2(b) and 2(c)). In Figure 2(b) in contrast to its activity in preventing liver-related diseases, including HCC, T_3_/TR may promote hepatoma cell metastasis and invasion. In earlier experiments, SCID mice receiving injections of 1 × 10^7^ J7-TR cells were randomly divided into hypothyroid, euthyroid, and hyperthyroid groups. All animals were sacrificed 4 weeks after cell injection, and lungs were removed for tumor biopsy. H&E-stained images from lungs of the corresponding mouse groups are shown in the bottom left panels. The metastatic index (average fold increase in the density of tumor foci in hypothyroid, hyperthyroid or euthyroid mice per cm^2^ of lung area) indicates that T_3_ promotes metastasis of TR-overexpressing hepatoma cells (bottom right panel). Thus, this study provided novel evidence that T_3_ influences aggressive tumor cell metastasis via TGF-*β* signaling [124].

Various proteolytic enzymes that play important roles in tumor invasion and metastasis processes have been identified. These proteases include cathepsins, plasmin, and plasminogen activators [131, 132]. Recently, employing cDNA microarrays combined with a quantitative proteomic approach, we identified and characterized T_3_-regulated proteins secreted by HepG2-TR*α*1 cells [133]. Several proteolytic enzymes involved in the urokinase plasminogen activator system, including plasminogen activator inhibitor-1 (PAI-1), urokinase receptor (uPAR), and PRSS22 (protease serine S member 22), were detected. The urokinase plasminogen activator system constitutes a family of serine proteases that includes urokinase-type plasminogen activator (uPA), uPAR, and PAIs [134]. This biological system has been implicated in diverse physiological and pathologic processes, including cell growth, migration, angiogenesis, inflammation, and metastasis [134]. Generally, the serine protease activity of uPA catalyzes the transformation of plasminogen (inactive) to plasmin (active). This process is involved in the degradation of the ECM and basement membrane, which is an important contributor to cancer cell metastasis. Previous reports have indicated that these processes are induced directly or indirectly through pro-MMP activation [132, 135]. uPAR is highly expressed in multiple types of cancers, including liver, colon, breast, lung, stomach, and ovarian cancers [136], and binds to uPA, pro-uPA, and the ECM protein, vitronectin, with high affinity; this leads to activation of the serine protease, uPA, thereby regulating proteolytic activity at the cell surface [137].

Activation of uPA and tissue plasminogen activator (tPA) is suppressed by the serine proteinase inhibitors, PAI-1 and PAI-2, with PAI-1 playing the predominant role [138]. PAI-1 rapidly associates with both uPA and tPA to modulate fibrinolytic activity in the vasculature. In particular, PAI-1 has been linked with the metastatic process, poor prognosis, and high mortality [134]. PRSS22, a component of the urokinase plasminogen activator system, acts as a serine protease to catalyze activation of uPA. Functional analyses have shown that PRSS22 mediates uPA activation and enhances the motility of smooth muscle cells through degradation of basement membrane-like ECM [139]. Thus, our previous findings collectively suggest that T_3_/TR enhances hepatoma cell metastasis via upregulation of members of the urokinase plasminogen activator system [133] (Figures 2(b) and 2(c)).

Among proteolytic enzymes, the cysteine cathepsin family member cathepsin H is also induced in TR-expressing hepatoma cells upon T_3_ treatment [140]. Cysteine cathepsins are a family of lysosomal proteases involved in normal cellular protein degradation and turnover. The majority of cathepsins are ubiquitously expressed in all human tissues. However, these proteases are often upregulated in various human cancers and have been implicated in distinct tumorigenic processes, such as angiogenesis and cell growth, death, and metastasis [141–143]. Given the involvement of cysteine cathepsin enzymatic activity in cancer progression, inhibition of these proteins may be an effective anticancer therapeutic strategy [144]. Previous experiments have revealed that cathepsin H is upregulated by T_3_/TR, leading to MMP or ERK activation and increased cell migration [140], consistent with the roles of ERK signaling in T_3_-induced migration (Figures 2(b) and 2(c)).

*β*-Catenin participates in multiple pathophysiological processes, including embryogenesis, tissue homeostasis, and tumor development [145]. *β*-Catenin is also a well-known mediator in the Wingless/Wnt signaling pathway [146]. Wnt proteins exert specific physiological functions by binding to frizzled receptors and low-density lipoprotein receptor-related proteins 5 and 6 (LRP5/6) to trigger canonical or noncanonical Wnt pathways [146]. *β*-Catenin accumulates in the early stages of HCC and plays a critical role in promoting tumor progression by stimulating tumor cell proliferation and reducing the activity of cell-adhesion systems. Consistent with this, *β*-catenin expression in HCC is correlated with poor prognosis, particularly in patients with poorly differentiated HCCs [147–149]. A recent study by our group showed that T_3_/TR inhibits the Wnt signal pathway via upregulation of Dickkopf (DKK)-4 [27], a secretory protein belonging to the DKK family, whose members act as antagonists of the Wnt signal pathway [27, 150]. Specifically, DKK4 was downregulated in 67.5% of HCC cancerous tissues (79 of 117), and its expression was accompanied by a concomitant decrease in TR protein levels compared with adjacent noncancerous tissues in matched cancerous tissues in 31% of cases (35 of 113). Furthermore, tissue array analyses have shown that TRs and DKK4 expression levels in normal and cancerous specimens are positively correlated. These data collectively indicate that T_3_/TR suppresses the Wnt/*β*-catenin pathway via DKK4 induction, leading to inhibition of hepatoma cell proliferation [27] (Figure 2(a)).

TNF-related apoptosis-inducing ligand (TRAIL), cloned on the basis of gene homology with DNA encoding the extracellular domain of tumor necrosis factor (TNF) and the CD95 ligand (Fas ligand, FasL), belongs to the TNF-*α* family [151, 152]. Investigations to date have focused on the ability of TRAIL to induce apoptosis in cancer cells. However, a few studies have additionally shown that TRAIL promotes not only apoptotic, but also nonapoptotic, pathways, including those involving protein kinase C (PKC), nuclear factor kappa B (NF-*κ*B), and MAPK [153]. The nonapoptotic signaling pathways triggered by TRAIL induce genes that promote cell growth, angiogenesis and metastasis, which contribute to cancer progression. Recently, T_3 _was shown to induce TRAIL in TR-expressing cells. However, TR-expressing hepatoma cells treated with T_3_ were apoptosis-resistant, even upon upregulation of TRAIL. This apoptosis resistance may be attributable to simultaneous upregulation of the Bcl-2 (B-cell lymphoma 2) family member, Bcl-xL (B-cell lymphoma-extra large), by T_3_. It has been proposed that TRs induce the expression of TRAIL, which acts in concert with simultaneously synthesized Bcl-xL to promote metastasis through nonapoptotic signals, such as NF-*κ*B and MAPK pathways [25] (Figures 2(b) and 2(c)).

Bcl-xL is a potent regulator of cell death and survival that not only contributes to tumor progression, but also confers cancer cell resistance to apoptosis triggered by conventional anticancer treatments [154–156]. Therapeutic strategies aimed at repressing apoptotic machinery by targeting Bcl-xL members may therefore have potential in cancer treatment [157, 158]. Consistent with previous suggestions that Bcl-xL acts as an anti-apoptotic factor in the context of chemotherapeutic treatment, T_3_ exhibits anti-apoptotic characteristics in hepatoma cells challenged with chemotherapeutic drugs whose actions are attributable to Bcl-xL induction [25] (Figure 2(c)).

Pituitary tumor transforming gene 1 (PTTG1) has been characterized as a human securin involved in cell-cycle regulation and sister chromatid separation during mitosis [159]. However, PTTG1 is highly abundant in several cancers and causes genome instability. Experiments by Cheng and colleagues using a mouse model harboring a TR*β* mutant (TR*β* ^PV/PV^) revealed that accumulated PTTG1 physically interacts with the DNA-binding domain of TR*β*1 through the N-terminal region. Subsequent experiments demonstrated that T_3_-bound TR*β*1 promotes proteasome activator 28c (PA28c)-mediated degradation of PTTG1 via association with steroid receptor coactivator 3 (SRC-3). In contrast, the TR*β*1PV mutant lacks ligand-binding ability and cannot interact directly with SRC-3/PA28c to activate proteasome-mediated degradation; as a result, PTTG1 accumulates [160]. Another study by our group revealed a different mechanism of PTTG1 suppression by T_3_/TRs, showing that T_3_ stimulation in TR-expressing hepatoma cells inhibited expression of specificity protein 1 (Sp1), thereby suppressing PTTG1, a direct transcriptional target of Sp1. Moreover, knockdown of Sp1 or PTTG1 inhibited proliferation of TR-expressing hepatoma cells [24]. These results collectively suggest that T_3_-bound TR*β*1 regulates the expression of PTTG1 via transcriptional and post-translational mechanisms to maintain regular cell-cycle control, whereas TR*β*PV induces aberrant accumulation of PTTG1, causing genetic instability during cancer development (Figure 2(a)).

In addition to crosstalk of TH signaling with other important pathways, T_3_ regulates metabolism-related genes or ECM proteins that influence liver cancer progression, such as Aldo-keto reductase family 1, member B1 (AKR1B1), Methionine Adenosyltransferase Ia (MAT1A), and Spondin 2 [26, 161, 162]. 

AKR1B1, a member of the AKR superfamily, participates in glucose metabolism and displays protective activity against toxic aldehydes derived from lipid peroxidation and steroidogenesis that affect cell growth upon accumulation [163, 164]. MAT1A is an essential liver enzyme that catalyzes the formation of S-adenosylmethionine (AdoMet), a methyl donor in cellular metabolism [165, 166]. Spondin 2 (also known as mindin) is a member of the mindin-F-spondin family of ECM proteins and functions as a pattern-recognition molecule for microbial pathogens. Consistent with this latter function, spondin 2-deficient mice exhibit severe impairment of neutrophil and macrophage recruitment upon microbial stimulation. These results highlight important roles of spondin 2 in mediating cellular inflammatory effects [167], and implicate TH in diverse functions that influence liver cancer progression.

## 4. Application of Thyroid Hormone Analogs in Liver-Related Disease Therapy

THs affect growth, metabolism, and physiological functions of nearly all organs [36]. Certain actions of THs are therapeutic, such as enhancing cardiac function, promoting weight loss, and reducing serum cholesterol. However, excess TH production by the thyroid gland or administration of exogenous THs may increase heart rate, potentially leading to atrial arrhythmia and heart failure, muscle wasting, and other symptoms [168]. During the past few decades, scientists have explored the possibility of identifying TH derivatives, with a view toward separating the beneficial actions of TH from its deleterious effects [29, 168–171]. These efforts have led to the identification of several derivatives of TH with potential therapeutic applications in the prevention or therapy of liver-related diseases.

GC-1, the first TR*β* agonist generated and a scaffold compound for the development of other TH derivatives, can be synthesized with higher efficiency and is more easily modified than native TH [172]. *In vitro* experiments have demonstrated that GC-1 displays a higher binding affinity to TR*β*1 than TR*α*1. In addition, GC-1 binds all major TR*β* isoforms with an affinity similar to that of T_3_. GC-1 preferentially accumulates in the liver, whereas its uptake is low in other tissues, including heart and skeletal muscle. This agonist selectively interacts with TR*β* within hepatocytes and exerts gene-specific activity comparable to that of native TH [168].

In an experimental rat model of CMD diet-induced NASH, T_3_ or GC-1 treatment has been shown to prevent steatohepatitis. Moreover, GC-1 had no significant side effects on heart rate; prevented and reversed CMD-induced fat accumulation in the liver; and ameliorated steatohepatitis [12]. These studies by Columbano and coworkers suggest that THs and TH analogs play significant roles in NASH prevention and therapy. These researchers additionally focused on investigating the effects of T_3_ and the selective agonist, GC-1, in an animal model of hepatocarcinogenesis. In rats treated with diethylnitrosamine (DEN) combined with a choline-deficient (CD) diet for 10 weeks, multiple preneoplastic lesions positive for glutathione S-transferase pi (GSTP) were observed. However, coadministration of T_3_ or GC-1 dramatically reduced GSTP-positive foci caused by DEN [120–122]. These experimental models provide strong evidence that TR agonists present effective targets in liver cancer therapy (Figure 2(a)).

KB-141 is a TH analogue that binds to human TR*β* with an affinity 14-fold higher than that for TR*α*. In cholesterol-fed mice, rats, and cynomolgus monkeys, KB-141 lowered plasma cholesterol levels and lipoprotein Lp (a) without eliciting deleterious cardiac effects [173]. In cholesterol-fed ob/ob mice, KB-141 displayed a similar reducing effect on serum cholesterol, nonesterified fatty acids (NEFA), and hepatic TG levels. In addition, intraperitoneal glucose tolerance tests revealed that KB-141 treatment improved glucose tolerance and insulin sensitivity in ob/ob mice [174]. Thus, the selective TR*β* agonist, KB-141, may be a useful candidate for attenuating the features of the metabolic syndrome.

KB2115 (Karo Bio) has been described as a TR*β*-selective agonist that is preferentially taken up by the liver. However, the extent to which the liver-specific distribution of KB2115 differs from that of native THs is unclear. Until recently, only clinical trials with KB2115 have been published [175, 176]; however, follow-up studies for this compound have been presented publicly (KaroBio website). In a clinical trial, oral administration of KB2115 daily for 2 weeks significantly lowered serum total cholesterol, LDL cholesterol, and apo B levels, and the ratio of apo B to apo A-I in humans with moderately increased cholesterol, compared with placebo [176]. Another multicenter trial performed to assess the safety and efficacy of KB2115 in lowering serum LDL cholesterol in patients with hypercholesterolemia who were already receiving statin showed that KB2115 lowered serum LDL cholesterol, TG, and Lp (a) levels in a dose-dependent manner in the presence of statins. These reductions in the presence of statin, which were of the same magnitude as those achieved by KB2115 alone, are clinically relevant and indicate that KB2115 is as effective in patients who are already on statin therapy as in those who are not [175].

Recently, Metabasis developed MB0781, a liver-selective prodrug. After hepatic first-pass extraction and tissue-selective activation via enzymatic cleavage, MB0781 is converted to MB07344, the active form. MB07344 is a strong TR*β* ligand and binds other TRs with significantly lower affinity. Its actions may thus be characterized as highly liver- and TR*β*-selective [94].

In animals with diet-induced obesity, treatment with MB07811 for only 2 weeks at doses that did not affect body weight or glycemia, or induce other side effects of the TH axis led to a significant reduction in total plasma cholesterol and both serum and hepatic TG levels [94]. MB07811 exerted similar effects in diabetic fatty mice and rats and markedly reduced hepatic steatosis as well as plasma FFA and TG levels (Figure 2(a)). Clearance of liver lipids by MB07811 may be attributable to changes in hepatic gene expression that lead to accelerated hepatic fatty acid oxidation, as evidenced by increased hepatic mitochondrial respiration rates [13].

Thus, selective targeting with TR agonists, such as GC-1, KB-141, KB-2115, and MB07811, may present an innovative strategy for treatment of liver-related diseases, such as metabolic diseases, simple hepatic steatosis, and HCC [12, 13, 94, 120, 175, 176].

## 5. Conclusions

The multipotent functions of THs and TRs are variable, but are essential for normal growth and proliferation of different tissues. The physiological functions of TH/TR are mediated through downstream genes induction by genomic or nongenomic actions (Table 1). Disruption of TH signaling causes dysfunction in several organs and is closely associated with several diseases [1–7, 9]. The liver is one of the major target organs of TH, and TH levels are closely correlated with multi liver-associated diseases within a spectrum ranging from hepatic steatosis to HCC [23, 111, 115]. Thus, the complex mechanism of TH action requires further investigation. In this article, we have discussed the growing complexity of TH signaling, and highlighted the advantages associated with TH-based therapeutic strategies for liver-related diseases as well as recent findings that may be exploited to improve therapeutic outcomes.

Considerable evidence supports the utility of TH administration as an effective strategy for prevention of liver-related diseases, including steatosis, hepatitis, and HCC [12, 13, 91, 92, 94, 171]. However, several challenges confront conventional TH-based cancer therapy, reflecting the variability and complexity of processes involved in cancer progression. Moreover, individual TR isoforms have different effects in different tumor types and stages of development. Signaling via T_3_ and its interacting partners may facilitate the switch from tumor suppression in premalignant stages of tumorigenesis to tumor promotion in later stages of liver cancer.

The adult liver is a quiescent organ that exhibits an extremely low replication rate (mitosis frequency < 1/20,000 hepatocytes). However, following the reduction of liver mass by partial hepatectomy, or chemical-, nutrition-, vascular-, or viral-mediated liver injury, hepatocytes begin to proliferate in a relatively synchronized manner [120, 177]. Several lines of evidence suggest that conditions characterized by liver damage and accelerated hepatocyte turnover rate (liver regeneration) are positively associated with the incidence of HCC [178–180]. However, instead of promoting liver growth with a typical pattern of liver regeneration, T_3_ has been reported to act as a hepatomitogenic factor to stimulate liver cell growth without associated cell loss/death. Moreover, experiments using the DEN-induced HCC animal model have shown that hepatocyte proliferation induced by T_3 _is associated with a decrease in HCC development [119, 121, 122]. By contrast, T_3_ not only reduced the size of hepatic preneoplastic nodules of rats suffering from HCC, it also suppressed aberrant cellular growth by controlling the expression of cell-cycle regulators, such as PTTG1, ppRb, CDK2, and cyclin E in hepatoma cell lines [24, 125, 160]. Thus, TH acts as a potent hormone to control organ homeostasis, including that of the liver. These studies further imply that T_3_ may foster hepatic homeostasis by controlling the growth of either adult hepatocytes or hepatic neoplasm; however, the various reported effects of TH on cellular proliferation suggest a dependence on the specific cell type and cellular microenvironment.

Although TH/TR arguably has suppressor functions in hepatocellular carcinogenesis, in apoptosis-resistant hepatoma cells, TH/TR promotes tumor cell metastasis by upregulating several ECM proteases, such as furin, cathepsin H, and MMP, via TRAIL signaling [25, 124, 140]. Further studies are required to establish the specific functions of TRs in different tumor development processes.

## Figures and Tables

**Table 1:** Summary of the relevant TH target genes in hepatocyte.

Physiological function	Gene name	Upregulated by TH/TR	Downregulated by TH/TR	Genomic action	Nongenomic action	Reference
Homeostasis control	Er*α*	●			●	[61]
	HIF-1*α*	●			●	[58, 68]

Ion transporter	KCNH2		●		●	[71]

Cellular metabolism	GLUT1	●			●	[68]
	PFKP	●			●	[68]
	MCT 4	●			●	[68]
	mGPDH	●		●		[13, 94]
	CPT-1	●		●		[13, 94]
	SREBP-1c	●		●		[13, 94]
	PPAR*α*	●		●		[95]
	PPAR*δ*		●	●		[95]
	PPAR*γ*	●		●		[95]
	ACOX	●		●		[95]
	SCD	●		●		[95]
	ATGL	●		●		[95]
	Apo B	●		●		[95]
	AKR1B1	●		●		[26]

Cellular death control	TRAIL	●		●		[25]
	Bcl-xL	●		●		[25]

Inflammation	COX-2		●	●		[12]
	STAT3		●	●		[12]

Cell growth control	TGF-*β*	●		●		[125]
	CDK2		●	●		[125]
	PTTG1		●	●		[24]

Cell metastasis	PAI-1	●		●		[133]
	uPAR	●		●		[133]
	PRSS22	●		●		[133]
	Cathepsin H	●		●		[140]
	DKK4	●		●		[27]
	MAT1A	●		●		[162]
	Spondin 2	●		●		[161]
